# Preoperative validation of edema-corrected tractography in neurosurgical practice: translating surgeon insights into novel software implementation

**DOI:** 10.3389/fneur.2023.1322815

**Published:** 2024-01-08

**Authors:** Sebastian F. Koga, Wesley B. Hodges, Hayk Adamyan, Tim Hayes, Peter E. Fecci, Vadim Tsvankin, Gustavo Pradilla, Kimberly B. Hoang, Ian Y. Lee, Eric W. Sankey, Patrick J. Codd, David Huie, Brad E. Zacharia, Ragini Verma, Vatche G. Baboyan

**Affiliations:** ^1^Franciscan Missionaries of Our Lady Health System, Baton Rouge, LA, United States; ^2^Synaptive Medical Inc., Toronto, ON, Canada; ^3^Department of Neurosurgery, Duke University Medical Center, Durham, NC, United States; ^4^Colorado Brain and Spine Institute, Englewood, CO, United States; ^5^Department of Neurosurgery, Emory University School of Medicine, Atlanta, GA, United States; ^6^Department of Neurosurgery, Henry Ford Health System, Detroit, MI, United States; ^7^Department of Neurosurgery, Penn State Hershey Medical Center, Hershey, PA, United States; ^8^Department of Radiology, University of Pennsylvania, Philadelphia, PA, United States; ^9^Cohen Veterans Bioscience, New York, NY, United States

**Keywords:** tractography, peritumoral zone, edema correction, fiber tracking, diffusion tensor imaging, free-water correction

## Abstract

**Background:**

Peritumoral edema alters diffusion anisotropy, resulting in false negatives in tractography reconstructions negatively impacting surgical decision-making. With supratotal resections tied to survival benefit in glioma patients, advanced diffusion modeling is critical to visualize fibers within the peritumoral zone to prevent eloquent fiber transection thereafter. A preoperative assessment paradigm is therefore warranted to systematically evaluate multi-subject tractograms along clinically meaningful parameters. We propose a novel noninvasive surgically-focused survey to evaluate the benefits of a tractography algorithm for preoperative planning, subsequently applied to Synaptive Medical’s free-water correction algorithm developed for clinically feasible single-shell DTI data.

**Methods:**

Ten neurosurgeons participated in the study and were presented with patient datasets containing histological lesions of varying degrees of edema. They were asked to compare standard (uncorrected) tractography reconstructions overlaid onto anatomical images with enhanced (corrected) reconstructions. The raters assessed the datasets in terms of overall data quality, tract alteration patterns, and the impact of the correction on lesion definition, brain-tumor interface, and optimal surgical pathway. Inter-rater reliability coefficients were calculated, and statistical comparisons were made.

**Results:**

Standard tractography was perceived as problematic in areas proximal to the lesion, presenting with significant tract reduction that challenged assessment of the brain-tumor interface and of tract infiltration. With correction applied, significant reduction in false negatives were reported along with additional insight into tract infiltration. Significant positive correlations were shown between favorable responses to the correction algorithm and the lesion-to-edema ratio, such that the correction offered further clarification in increasingly edematous and malignant lesions. Lastly, the correction was perceived to introduce false tracts in CSF spaces and - to a lesser degree - the grey-white matter interface, highlighting the need for noise mitigation. As a result, the algorithm was modified by free-water-parameterizing the tractography dataset and introducing a novel adaptive thresholding tool for customizable correction guided by the surgeon’s discretion.

**Conclusion:**

Here we translate surgeon insights into a clinically deployable software implementation capable of recovering peritumoral tracts in edematous zones while mitigating artifacts through the introduction of a novel and adaptive case-specific correction tool. Together, these advances maximize tractography’s clinical potential to personalize surgical decisions when faced with complex pathologies.

## Introduction

For several decades, *in vivo* white matter dissections made possible by diffusion-weighted MRI (dMRI) and fiber tractography algorithms have been used routinely as technological adjuncts when treating brain tumors (1). In the presence of aggressive brain malignancies, however, the utility of tractography in surgical planning is significantly challenged by the dMRI signal dilution caused by pathology-induced tract invasion and vasogenic edema (2, 3) which compromise accurate tract reconstructions in clinically significant areas of interest; namely, at the brain-tumor interface and the broader edematous zone (4). The clinical implementation of advanced diffusion modeling to more accurately characterize the subcortical anatomy in the presence of complex oncological lesions is motivated by the finding that maximizing extent of resection improves survival insofar as neurological deficits are mitigated during surgery (5). With histopathological studies demonstrating the presence of tumor cells outside of contrast-enhancing margins located within the fluid-attenuated inversion recovery (FLAIR) signal abnormality (2, 6, 7), and with the understanding that gliomas (but not metastases) migrate along white matter tracts (8–11), improved neuroimaging of subcortical structures is essential. Interestingly, direct electrical stimulation (DES) mapping studies have also reported the presence of positive sensorimotor and language sites located *within* intratumoral margins of gliomas to further underscore the need to preoperatively assess the white matter integration patterns of the glioma-network interface (12). Although efforts have focused on resolving heterogeneous fiber populations using crossing fiber models with advanced dMRI acquisition protocols (13–17), resolving the issue of tractography-susceptible edematous voxels is needed both in clinically realistic acquisitions and with commercially available implementations for maximum broader impact (15, 18, 19).

If diffusion weighted tractography is to evolve as a standard of care in neurosurgical oncology, diffusion signal modeling proximal to pathological brain tissue must improve to better account for the true signal characteristics of the underlying white matter therein. Recent efforts have attempted to address this problem - specifically in clinically feasible (single-shell) DTI acquisitions (18) - by fitting bi-compartmental models that estimate the free-water present at every voxel while also modeling tissue anisotropy (15, 18–20). Such free-water correction (FWC) efforts intend to improve the tractography process by correcting the diffusion signal in voxels impacted by edema. Indeed, recent intraoperative motor mapping studies have reported better anatomical correspondence between positive DES sites and white matter tract reconstructions generated by a free-water corrected tractography algorithm called FERNET (18, 21).

In spite of these research breakthroughs, the field lacks (1) a systematic preoperative evaluation paradigm to derive clinically meaningful insights of advanced tractography algorithms beyond what is reported by DES analyses of individual tracts and (2) a commercially available implementation of free-water corrected tractography that flexibly accommodates pathologies with varying edematous zones (18, 21). The primary surgical indication for the use of this adjunct is to aid in the selection of an optimal parafascicular trajectory when facing lesions that engage eloquent white matter structures and minimize the likelihood of iatrogenic injury thereafter. Although DES remains the gold standard for intraoperative functional mapping and guidance of resection strategy, its preoperative unavailability precludes its utility in neurological risk assessment, patient counseling, and trajectory planning. Moreover, a qualitative radiographic assessment tool to validate multi-subject tractograms along clinically meaningful parameters is warranted particularly aiming to detect subcortical effects such as white matter tract displacement (2, 22), tract infiltration (2, 12, 23, 24), and tract reduction (2, 19). In this study, we present a novel and noninvasive subcortical tractogram assessment survey to evaluate the “edema-invariant” FERNET tractography algorithm for neurosurgical planning. Ten surgeons specialized in neuro-oncology were prompted to assess various properties of the peritumoral environment when selecting the optimal surgical approach in patient datasets featuring heterogenous histological lesions with varying edematous zones. In deriving clinician insights from this survey, a commercially available software implementation is introduced to facilitate a practical integration of FWC into the neurosurgical workflow.

## Materials and methods

### Patients and study design

**Figure 1:**
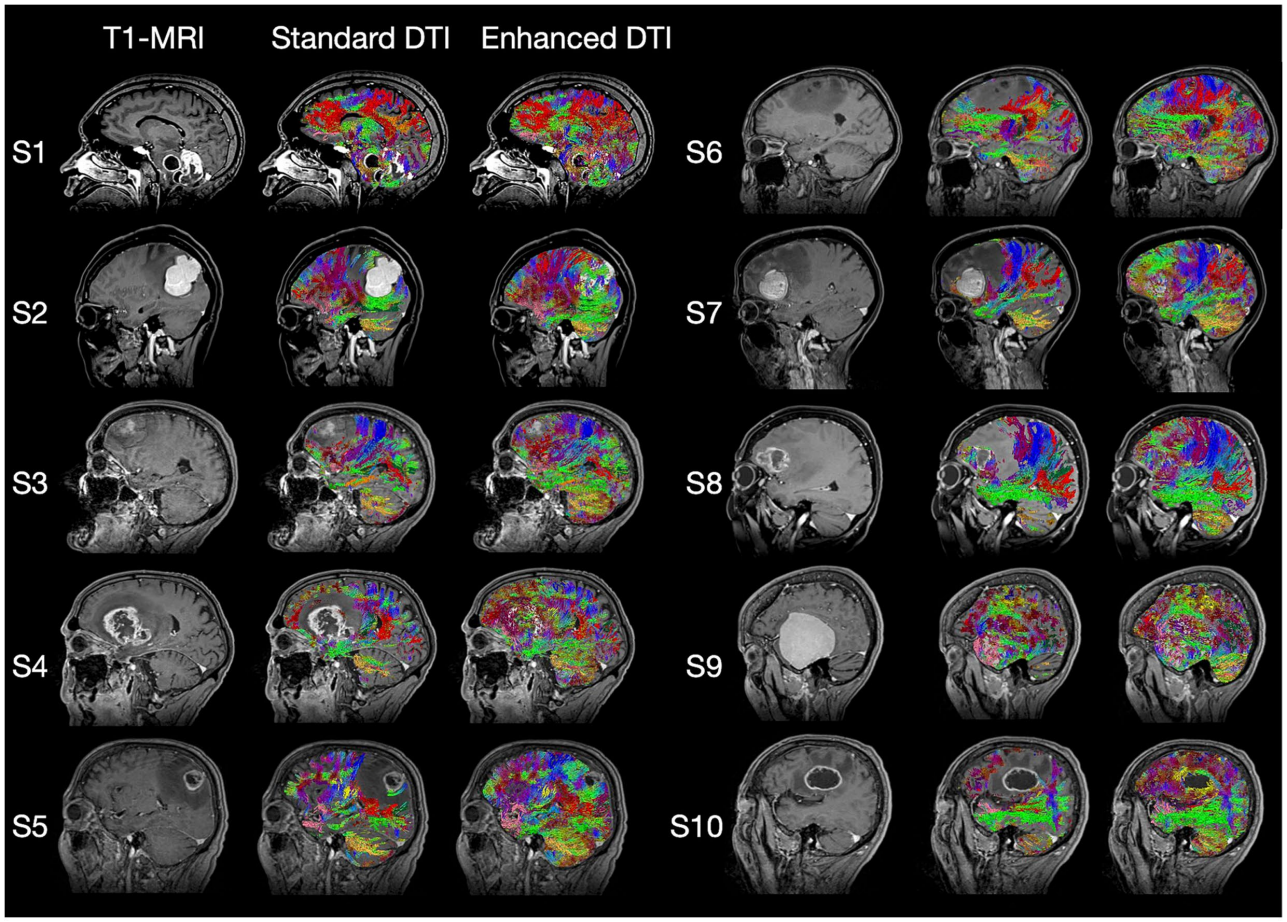
Standard T1-weighted MRI, Standard DTI Tractography, and Enhanced DTI Tractography neuroimages for each of the 10 subjects (i.e., patients) evaluated. Tract recovery profiles with enhanced DTI (Columns 3 and 6) will vary depending on the associated intra- and peritumoral dynamics of the underlying pathology, with each tumor type featuring distinctly varying degrees of tract invasion, mass effect, necrosis, and peritumoral edema impacting the recovery process.

The imaging data from ten patients previously treated for oncological neurosurgery at Ochsner Medical Center (Table 1) were selected by their surgeon (S. Koga) on the basis of clinical and imaging findings demonstrating varying degrees of pathology-associated intracranial edema evident on structural MRI (Figure 1). MRI data were anonymized in accordance with Health Insurance Portability and Accountability Act (HIPAA) guidelines.

**Table 1:** Demographics and histology.

Subject ID	Age	Sex	Histopathological diagnosis
01	29	Male	Pilocytic astrocytoma (Anaplastic) IDH wild type (WHO III)
02	41	Female	Hemangiopericytoma (WHO II)
03	75	Female	Amyloid angiopathy
04	74	Male	Glioblastoma IDH wild type (WHO IV)
05	76	Male	Metastatic melanoma (skin)
06	53	Male	Metastatic adenocarcinoma (lung)
07	57	Female	Metastatic neuroendocrine carcinoma (lung)
08	70	Female	Glioblastoma IDH wild type (WHO IV)
09	73	Female	Meningioma (WHO I)
10	71	Male	Glioblastoma IDH wild type (WHO IV)

IDH, isocitrate dehydrogenase.

### MRI imaging analysis

The preoperative MRI protocol was acquired on a 1.5 T Siemens MAGNETOM Aera scanner (Siemens Healthcare) to include high-resolution, 3D magnetization-prepared rapid acquisitions with gradient echo (MPRAGE) with and without contrast-enhancement (TE = 3.32 ms, TR = 2,370 ms, TI = 1,430 ms, pixel resolution = 1 mm x 1 mm, slice thickness = 2 mm, flip angle = 8 degrees, FOV = 22.4 cm × 25.6 cm). DTI was acquired with 20 directions (b = 1,000 s/mm^2^, pixel size = 2 mm, slice thickness = 3 mm).

Neuroimages were processed using commercially available surgical planning software (Modus Plan, Synaptive Medical, Toronto). Modus Plan was modified to toggle the visualization of uncorrected and corrected whole-brain deterministic tractography. The latter was achieved by integrating a modified, licensed version of the Freewater estimatoR using the iNtErpolated iniTialization (FERNET) algorithm (developed by Verma and colleagues (19) through a Sponsored Research Agreement between Synaptive and the University of Pennsylvania) into Modus Plan’s processing pipeline to enable direct intra-subject comparisons between the datasets and qualitative assessment by each rater.

To assess the correlations between survey responses and histopathology, lesions and perilesional (i.e., non-enhancing) free-water maps were manually delineated on contrast-enhanced T1s and apparent diffusion coefficient (ADC) maps, respectively. To quantify the extent of free-water, the following ratio was calculated: Volume of abnormal *perilesional* voxels (evident on ADC) divided by the volume of total abnormal voxels (lesion + edema).

### Surgical tractography examination and aims

Fly-through video recordings were presented in three hierarchical stages: (Section 1) visualization of pathology on T1 scans (without overlaid tracts), (Section 2) visualization of pathology with standard (uncorrected) tractography, and (Section 3) visualization of the pathology with enhanced (corrected) tractography. At each stage of the recording, the survey items (Table 2) are narrated by a surgeon and audibly presented to the raters, with them being prompted to respond with their level of agreement (True/Partially True/False).

**Table 2:** Survey items, responses, and inter-rater reliability statistics for standard and enhanced DTI tractography datasets.

Section 2: Standard DTI survey items	UN-corrected tractography				Inter-rater reliability (IRR)				
	True (%)	Partially true (%)	False (%)	Ratings (N)	Agreement %	Chance agreement %	AC2 coefficient (SE)	Value of *p*	IRR rating
Brain-tumor interface: the scan demonstrates a clear interface between brain and pathological tissue	36 (42.35%)	24 (28.24%)	25 (29.41%)	85	63.64%	61.89%	0.05 (0.08)	0.566	Poor
False signal: false tracts are seen in sulci or ventricles (CSF spaces)	13 (15.29%)	21 (24.71%)	51 (60%)	85	70.84%	52.23%	0.39 (0.13)	0.015	Fair
False negative: tracts are missing in significant areas of the normal brain	11 (13.1%)	24 (28.57%)	49 (58.33%)	84	69.24%	53.70%	0.34 (0.1)	0.009	Fair
False negative: tracts are missing in the vicinity of the lesion	58 (69.05%)	12 (14.29%)	14 (16.67%)	84	85.33%	43.29%	0.74 (0.14)	<0.001	Substantial
Grey-white matter interface: the tracts appear to begin at the grey-white matter interface	51 (60.71%)	31 (36.9%)	2 (2.38%)	84	80.35%	46.16%	0.64 (0.04)	<0.001	Substantial
The scan aids spatial orientation compared to single axis images	65 (76.47%)	10 (11.76%)	10 (11.76%)	85	73.95%	35.41%	0.6 (0.09)	<0.001	Moderate
This scan demonstrates the following anatomical effects: tract reduction	52 (61.9%)	18 (21.43%)	14 (16.67%)	84	76.03%	49.78%	0.52 (0.17)	0.013	Moderate
This scan demonstrates the following anatomical effects: tract displacement	64 (75.29%)	17 (20%)	4 (4.71%)	85	81.60%	37.56%	0.71 (0.09)	<0.001	Substantial
This scan demonstrates the following anatomical effects: tract infiltration	26 (30.59%)	26 (30.59%)	33 (38.82%)	85	66.79%	62.69%	0.11 (0.12)	0.375	Poor
Surgical pathway: the optimal surgical pathway to the ROI is clear to me	57 (67.06%)	25 (29.41%)	3 (3.53%)	85	84.48%	45.91%	0.71 (0.11)	<0.001	Substantial

Section 3: Enhanced DTI survey items	Free-water corrected tractography				Inter-rater reliability (IRR)				
	True (%)	Partially true (%)	False (%)	Ratings (N)	Agreement %	Chance agreement %	AC2 coefficient (SE)	Value of *p*	IRR rating
Grey-white matter interface: the tracts appear to begin at the grey-white matter interface	53 (62.35%)	17 (20%)	15 (17.65%)	85	64.26%	51.97%	0.26 (0.09)	0.017	Fair
Lesion: definition of the lesion is further clarified	36 (42.35%)	23 (27.06%)	26 (30.59%)	85	60.14%	61.51%	−0.04 (0.08)	0.67	Poor
Brain-tumor interface: the interface between brain and pathological tissue is further clarified	34 (40%)	30 (35.29%)	21 (24.71%)	85	68.24%	61.87%	0.17 (0.12)	0.198	Poor
False signal: false tracts are seen in sulci or ventricles (CSF spaces)	39 (46.99%)	34 (40.96%)	10 (12.05%)	83	70.95%	56.40%	0.33 (0.09)	0.006	Fair
This scan demonstrates the following anatomical effects: tract reduction	27 (32.53%)	23 (27.71%)	33 (39.76%)	83	61.26%	61.80%	−0.01 (0.08)	0.866	Poor
This scan demonstrates the following anatomical effects: tract displacement	67 (78.82%)	15 (17.65%)	3 (3.53%)	85	83.72%	33.19%	0.76 (0.08)	<0.001	Substantial
This scan demonstrates the following anatomical effects: tract infiltration	44 (52.38%)	15 (17.86%)	25 (29.76%)	84	67.10%	56.23%	0.25 (0.17)	0.172	Fair
Surgical pathway: the optimal surgical pathway to the ROI is further clarified	31 (36.9%)	25 (29.76%)	28 (33.33%)	84	62.29%	62.56%	−0.01 (0.1)	0.941	Poor
Surgical pathway: the optimal surgical pathway is less clear	6 (7.14%)	17 (20.24%)	61 (72.62%)	84	83.82%	38.17%	0.74 (0.11)	<0.001	Substantial

DTI, Diffusion Tensor Imaging; AC2, Gwet’s 2nd Order Agreement Coefficient.

Items from Section 2 (of Table 2) were designed to assess the quality of standard tractography data as a baseline (aim 1) before progressing to the added benefits of enhanced tractography in Section 3 (aim 2):

- Tracts appear to begin at the grey-white matter junction
- Tracts are missing in significant areas of the normal brain (False Negatives)
- Tracts are missing in the vicinity of the lesion (False Negatives)

Next, we assessed the added clinical benefits of enhancing tractography with FWC when planning the optimal surgical approach (aim 2). To this aim, the five following prompts from Section 2 (*standard tractography assessment*) were repeated (i.e., paired) in Section 3 (*enhanced tractography assessment*):

- Tracts appear to begin at the grey-white matter interface
- False tracts are seen in sulci or ventricles (i.e., False Positives in CSF spaces)
- This scan demonstrates the following anatomical effects: Tract Reduction
- This scan demonstrates the following anatomical effects: Tract Displacement
- This scan demonstrates the following anatomical effects: Tract Infiltration

To assess false positives, raters were explicitly instructed to evaluate the presence of streamlines extending into the sylvian fissure, ventricular systems, or perimesencephalic cisterns. Lastly, raters directly assessed the added clarity provided by enhanced tractography when evaluating perilesional microarchitecture:

- The definition of the lesion is further clarified
- The interface between brain and pathological tissue is further clarified
- The optimal surgical pathway to the region of interest is further clarified

The video recordings and survey items for each of the ten patients were shared with the surgeon raters for scoring.

### Data analysis

The responses to each of the three survey sections were scored *(False = 1, Partially True = 2, True = 3)* and imported into RStudio for subsequent analysis (version 3.6.1, RStudio, Inc.). To assess rater confidence for each item, inter-rater reliability (IRR) was calculated using Gwet’s “second-order agreement coefficient” (Gwet AC2) with ordinal weighting applied (25). This IRR metric is robust in the presence of missing data and when high agreement is observed among multiple raters (>2), and therefore offers more stable IRR coefficients when compared to conventional metrics. We report the agreement percentages along with respective Gwet AC2 coefficients and value of *p*s associated with each questionnaire item. The Gwet AC2 coefficients reflect the extent of agreement among the raters and here they are interpreted with respect to the benchmark criteria developed and set forth by Richard and Koch (26): ≤0.20: poor, 0.21 to 0.40: fair, 0.41 to 0.60: moderate, 0.61 to 0.80: substantial, and 0.81 to 1.0: excellent reliability.

To compare the four matched paired survey items from Sections 2 and 3 (of Table 2), the Stuart-Maxwell Marginal Homogeneity Test (or generalized McNemar’s *χ*^2^ test for *k x k* contingency tables) was performed to evaluate the difference in response probabilities between the conditions – testing the null hypothesis that the response probabilities for the respective survey items were indistinguishable between sections. This test was performed using the *DescTools* package library in R (DescTools Version: 0.99.29) and resulting value of ps were corrected for multiple comparisons. Nonparametric spearman’s correlations were computed between the response rates in the survey items evaluating the added clarity provided with corrected tractography and the free-water ratios using the *stats* package library in R (stats version 3.6.1).

## Results

Of the 10 total surgeon raters enrolled, 7 completed the survey for all 10 datasets and 3 completed 5 out of 10. The response rates and responses to each of the survey items are listed in Table 2, along with their respective interrater reliability (IRR) statistics.

### Standard tractography assessment

**Figure 2:**
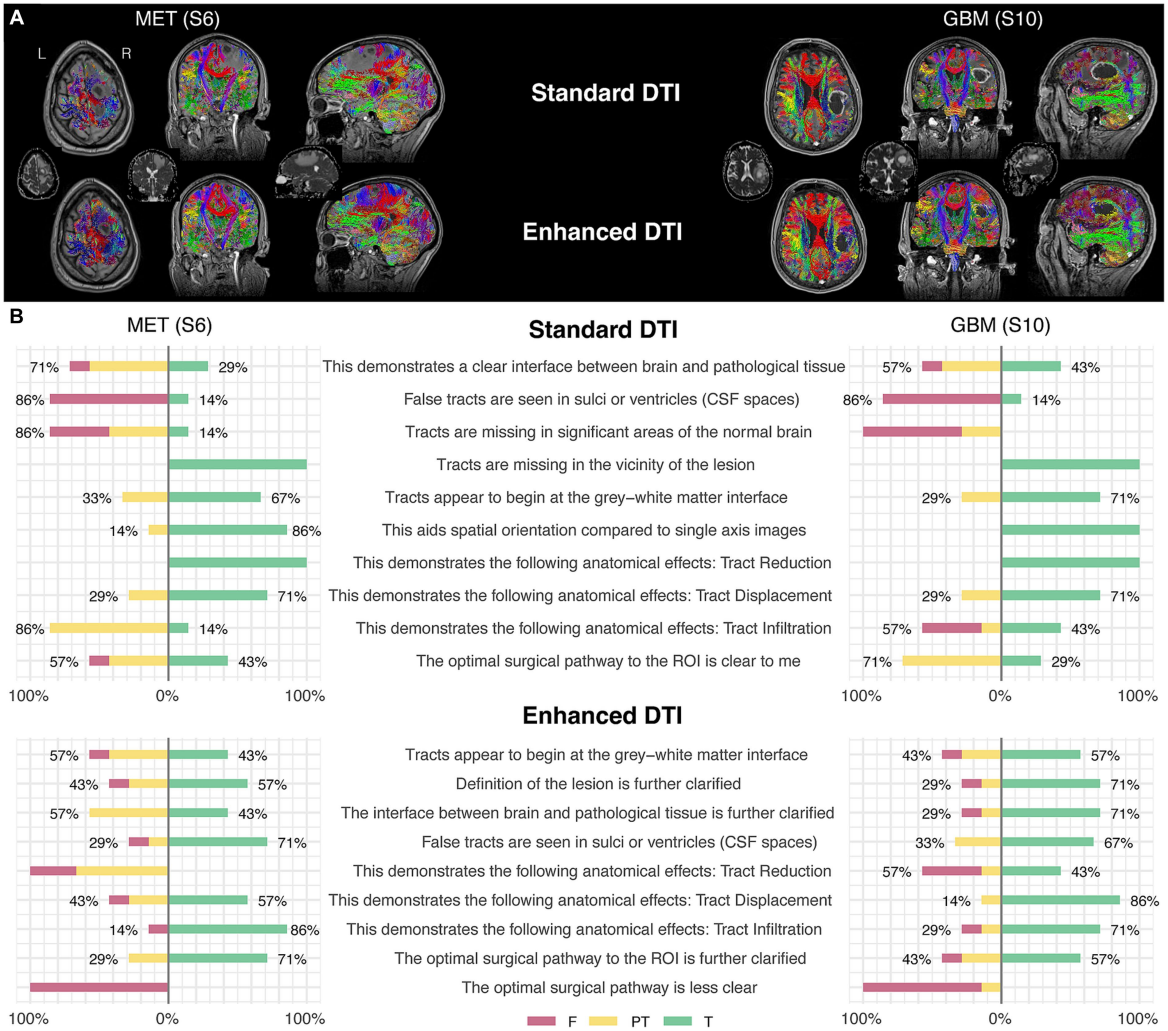
Two illustrative case examples and their respective survey scoring evaluations. **(A)** Two case examples of pathology-induced edema resulting in false negatives in the standard DTI tractograms (top row) impacting commissural (red), projection (blue), and association fascicles (green/yellow) in the peritumoral zone and the subsequent tract recovery with enhanced DTI (bottom row). Tractography-susceptible edematous zones are also shown on the respective ADC maps for each case (middle row). **(B)** Survey scoring from the two case examples corresponding to the images shown in the upper panel **(A)**, rated by the surgeon participants enrolled in the study.

When assessing the quality of conventional tractography data (Section 2, Table 2), substantial agreement was observed to the item, “tracts are missing in the vicinity of the lesion,” with the raters responding “True” in 58/84 (69%) of instances (AC2: 0.74, *p* < 0.001). This indicates that the conventional DTI data presents with considerable false negatives in areas surrounding the pathology (Figure 2; Table 2). Opinions otherwise relating to general data accuracy were favorable, with substantial agreement to the statement pertaining to tracts beginning at the grey-white matter interface (False: 2%, Partially True: 37%, True: 2%; AC2: 0.64, *p* < 0.001), and moderate agreement that the 3D rendering aided spatial orientation compared to single-axis images (True: 76%, AC2: 0.6, *p* < 0.001). Similarly, raters showed moderate and substantial agreement when prompted that the images demonstrated tract reduction and displacement, respectively (Tract Reduction: 62% True, AC2: 0.52, *p* < 0.05; Tract Displacement: 75% True, AC2: 0.71, *p* < 0.001).

When assessing tract infiltration, raters showed poor agreement with mixed responses weighted nearly equally (AC2: 0.11, *p* = 0.375). Lastly, raters substantially agreed that the optimal surgical pathway was clear (False: 3%, Partially True: 29%, True: 67%; AC2: 0.71, *p* < 0.001).

### Enhanced tractography assessment

When assessing the quality of enhanced tractography data (Section 3, Table 2), the majority of raters fairly agreed that the tracts appear to begin at the grey-white matter interface (62.35% True, AC2: 0.26, *p* < 0.05), but also that false tracts were seen in CSF spaces (12% False, AC2: 0.33, *p* < 0.01).

**Figure 3:**
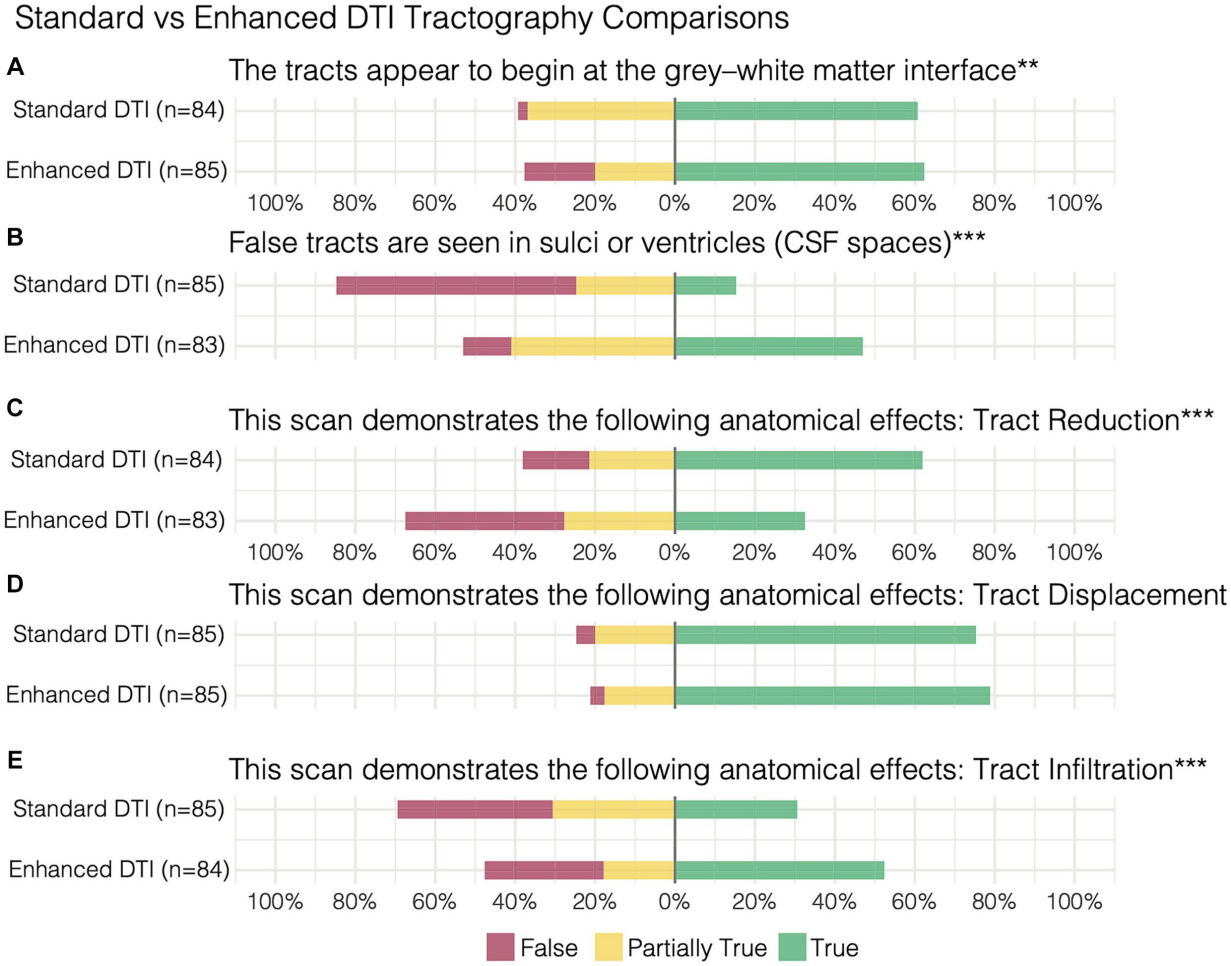
Paired survey items and results comparing standard vs. enhanced tractography assessment of **(A)** the tracts appearing at the grey-white matter interface, **(B)** false tracts appearing in CSF spaces in addition to demonstration of **(C)** tract reduction, **(D)** displacement, and **(E)** infiltration. **p < 0.01, ***p < 0.001.

When comparing the matched items, significant differences in response probabilities were observed when assessing tract reduction and infiltration, as standard tractography data showed tract reduction (62% True) and responses declined to 32% with the enhanced data [*χ*^2^ (dof) = 21.38(2), *p* < 0.001, corrected] (Figures 3C,E). Moreover, significant differences were observed regarding the assessment of tract infiltration between the datasets, with 31% rating this as true with the standard tractography which increased to 52% with enhanced tractography [χ^2^ (dof) = 18.01(2), *p* < 0.001, corrected] (Figure 3E). Interestingly, significant differences in responses were also observed when assessing tract terminations at the grey-white matter interface, such that the false responses in the standard tractography condition (2%) increased in the enhanced condition (18%) [*χ*^2^ (dof) = 13.62(2), *p* < 0.01, corrected]. When evaluating whether false tracts were seen in sulci or ventricles (i.e., CSF spaces), true responses increased from 15% in the standard tractography condition to 47% in the enhanced condition [*χ*^2^ (dof) = 38.93(2), *p* < 0.001, corrected].

In contrast, the assessments on the presence of tract displacement were not significantly different between the datasets [χ^2^ (dof) = 0.81(2), *p* = 1, corrected], indicating that tractography data - irrespective of free-water correction – is effective at demonstrating pathology-induced tract displacement (Figure 3D). When prompted with “the optimal surgical pathway was less clear,” respondes favored enhanced tractography with substantial agreement that this statement was false (False 73%, AC2: 0.74, *p* < 0.001).

**Figure 4:**
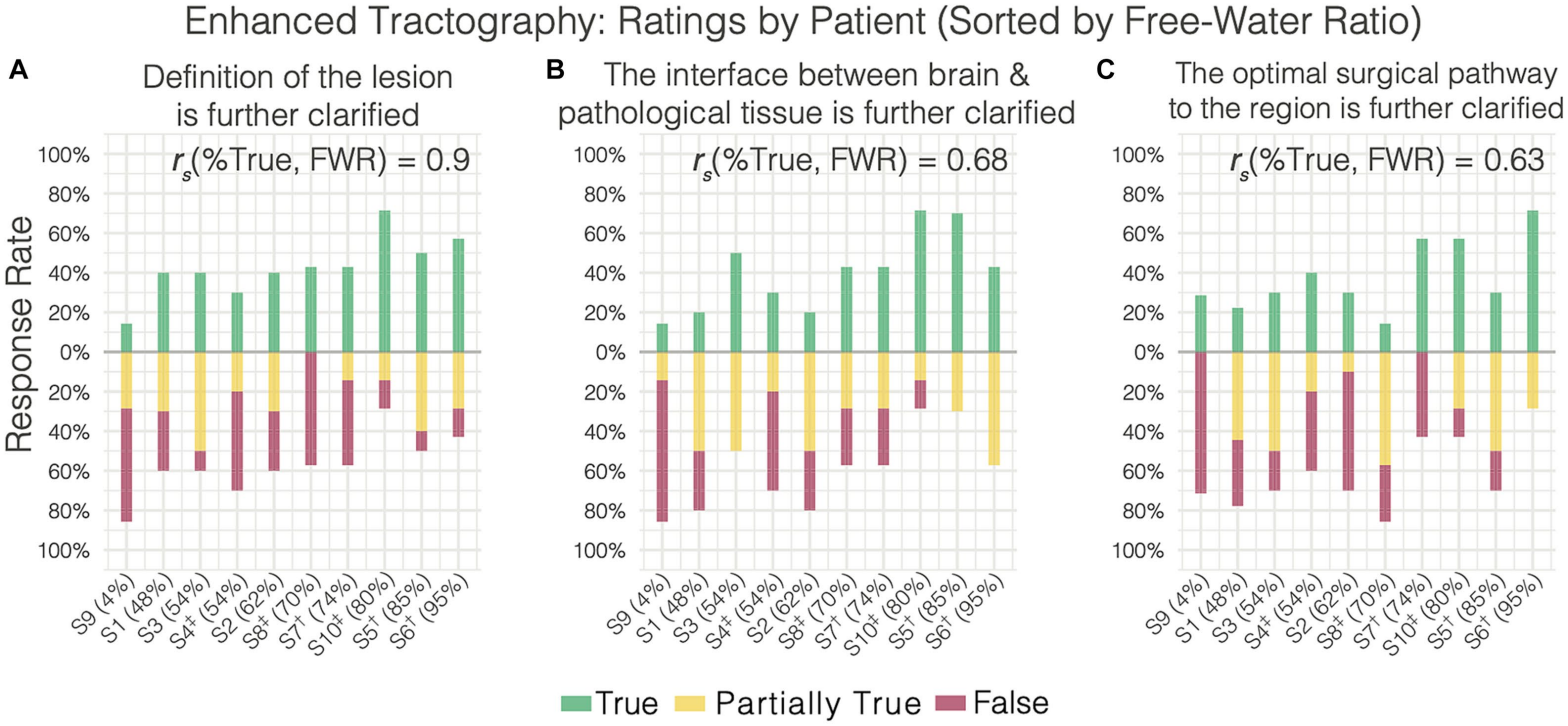
Enhanced tractography survey responses on individual patients (sorted increasingly by free water ratio) relating to further clarification of the **(A)** lesion definition, **(B)** brain-tumor interface, and **(C)** the optimal surgical pathway. Free water ratio was expressed as a percentage of abnormal perilesional voxels (evident on ADC) divided by the total abnormal voxels (lesion + edema). Nonparametric spearman’s correlations between the free water ratio and rate of true responses are shown for each item. ^†^Cerebral Metastases. ^‡^Glioblastoma.

When surveying the further clarification provided by enhanced tractography, rater responses were highly variable – resulting in poor agreement across the three items surveyed. This was driven by the variation in responses being associated with variations in perilesional free-water severity, such that the rates of “True” (i.e., favorable) responses to the enhanced data were positively correlated with the free-water ratio present across the datasets (Figure 4). For instance, patient 9 presented with a non-edematous meningioma and the majority of raters answered “False” on these survey items (i.e., further clarification was not added with FWC). Conversely, patient 6 presented with a highly edematous lesion (metastatic adenocarcinoma) and the majority of raters answered with “True.” When evaluating the lesion definition and brain-tumor interface, significant positive correlations were observed between the True response rate and free-water ratio (*r*_s_ = 0.9, *p* = 0.0004; *r*_s_ = 0.68, *p* = 0.0315, respectively). This positive correlation was also observed when evaluating the optimal surgical pathway (*r*_s_ = 0.6278, *p* = 0.052).

### Spurious tracts and adaptive correction

**Figure 5:**
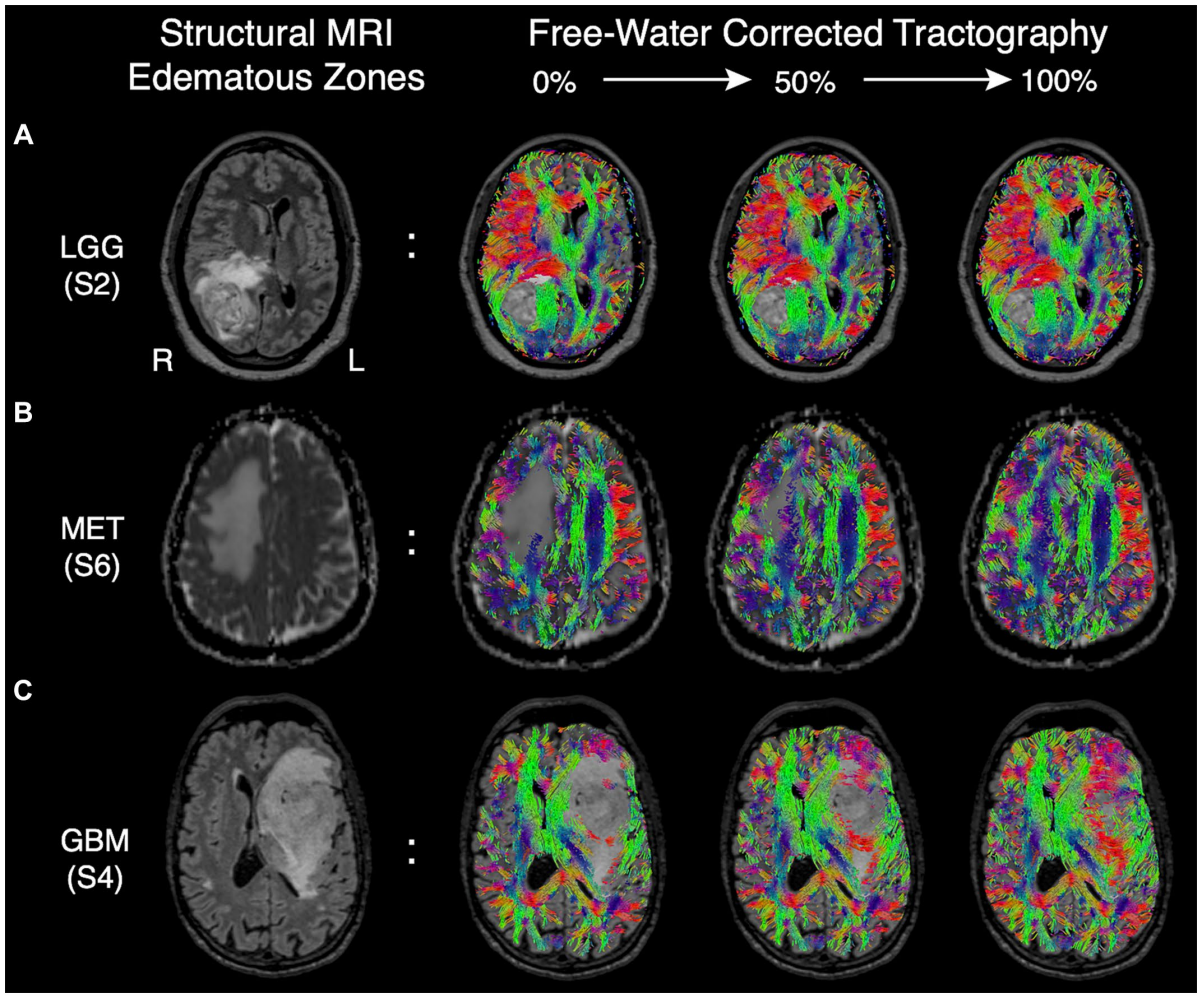
Edematous zones of 3 illustrative cases on structural MRI (Column 1) with subsequent recovery of false negative tracts using the dynamic free-water correction slider (Columns 2–4). The adaptive slider allows the user to control the desired level of tract recovery along a continuum of thresholds ranging from 0% (uncorrected, column 2) to 100% (fully corrected. Column 4). **(A)** Edematous zone of a low-grade (WHO grade II) glioma shown on T2 FLAIR, with progressive tract recovery delineating lesion-definition and mass effect on fibers in sagittal stratum of sachs. **(B)** Edematous zone of a metastatic adenocarcinoma shown on ADC, with progressive tract recovery within areas of vasogenic edema and subsequent midline shift (tract displacement) of frontal projection fibers but without tract invasion. **(C)** Edematous zone of a glioblastoma IDH-wild type shown on T2 FLAIR, with progressive tract recovery demonstrating tract infiltration (disruption) of fronto-temporal association fascicles, improved lesion definition, and mass effect (tract displacement).

Although FWC-enhanced data offered positive insight into the optimal surgical approach, showed less tract reduction, and improved assessment of tract infiltration (Figures 3C,E), these benefits came at the cost of increasing artifacts in CSF spaces and around the grey-white matter interface (Figure 3B). This finding resulted in a modification of Synaptive’s software implementation to minimize spurious tract reconstructions via free-water-parameterizing the tractography dataset, whereby each segment of the tractogram was encoded by the free-water map which assigns a normalized value representing the ‘degree of free water’ present along the segment. With the observation and understanding that spurious tract segments within CSF spaces are maximally occluded by free-water, the software’s graphical user interface was redesigned to enable dynamic thresholding of the tractogram to constrain tract recovery to non-CSF areas with higher concentrations of free-water (Figure 5). The adaptive thresholding method is demonstrated in the varying edematous zones of 3 illustrative cases: at its minimum value, FWC is un-applied and false negatives are observed (column 2), and at its maximum FWC is fully applied and tracts are completely recovered (column 4). With progressive increases to the correction slider, sub-threshold tract segments are dynamically recovered and visualized in real-time to facilitate inspection of subcortical effects regarding the lesion definition, tract infiltration, and mass effect (displacement). This individualized approach adapts to the surgeon’s intuition regarding the pathological and patient-specific effects impacting tracts within the peritumoral edematous zone (US patent No. 11,355,230) (27).

## Discussion

Given the implications of non-enhancing tumor (NET) cells on recurrence, surgical resection strategies have evolved to extend into the NET areas of the so-called “peritumoral zone” (PTZ) with substantial survival benefits being reported in patients with primary malignancies (16, 28). The ability to image this tractography-susceptible PTZ is therefore crucial not only to the assessment and mitigation of neurological risk preoperatively, but also to facilitate onco-functional decisions intraoperatively through visualization and preservation of eloquent white matter tracts when resections extend into these areas (i.e., for supratotal resections) (29, 30). In the case of secondary tumors such as brain metastases, they present with distinct extracellular free-water patterns which have been leveraged as features in their differentiation from glioblastomas (31–34). Although meningiomas are benign tumors whose growth is often contained in a tumor capsule (35), the perilesional brain is similarly affected by gliosis and sometimes by tumor invasion. Taken together, diffusion tractography has the unique potential to provide a noninvasive radiographic assessment of white matter tract alteration profiles caused by various neoplasms (2, 17, 22–24). In the present study, we demonstrate the perceived clinical potential of enhancing tractography within edematous voxels by showing that tract recovery improves assessment of both the brain-tumor interface and lesion definition while further clarifying the optimal surgical approach primarily in cases with brain malignancies (metastases and glioblastoma).

### Lesion definition and brain-tumor interface

The PTZ plays a crucial role in advancing our understanding of brain neoplasms, their behavior, and the development of targeted treatment strategies. With meningiomas typically encapsulated, the PTZ has been characterized by a well-defined border with inflammatory and compressive effects on surrounding brain tissue which frequently impacts the presence of vasogenic edema especially with larger tumor volumes (35). Gliomas, including both low- and high-grade, have been characterized as diffuse infiltrative diseases proliferating primarily along white matter tracts (2, 28, 36). Consequently, the glioma PTZ consists of infiltrative tumor cells nested within the edematous zone (23), a disruption to white matter architecture (28), and equivocal tumor margins beyond what is observed on conventional imaging. For cerebral metastases, the PTZ has been shown to be highly irregular depending on the primary tumor origin (32, 33), with more recent reports casting doubt on whether a sharp delineation from surrounding parenchymal tissue exists given the tumor invasion patterns commonly observed in histological samples further complicating its differentiation from glioblastomas (23) while potentially explaining the higher rates of recurrence. Moreover, vascular disruption to the blood–brain-barrier is strongly associated with significant increases in vasogenic edema within the metastatic PTZ (23), in contrast to infiltrative edema observed in the PTZ in gliomas (33).

Here, we have shown that enhancing tractography with FWC improved the visualization of subcortical anatomy in peritumoral areas with edema by recovering and delineating tracts at the brain-tumor interface (Figures 1, 4B). We’ve also shown that standard tractography is effective at evaluating tract displacement, but is problematic due to the presence of pathology-induced tract reduction (Figures 3C,D; Table 2). In contrast, enhancement unveils neuroanatomical data at the lesional interface and broader edematous zone evidenced by the poor agreement regarding responses in tract reduction apparent in corrected tractography (Table 2). This allowed surgeons to then qualify subcortical mass effect (tract displacement) which resulted in significantly increased capacity for assessment of white matter tract infiltration (Figure 3E; Table 3). These previously unavailable subcortical insights may subsequently be used in a variety of applications ranging from the quantification of disease progression or recovery after surgery, functional correlations, or serve as radiographic biomarkers especially when differentiating glioblastoma from cerebral metastases (31, 33, 34). Although responses were variable in assessment of the lesion definition and brain tumor-interface (Table 2), these items were affected by the amount of edema, such that benefits were more pronounced in highly edematous datasets consisting primarily of cerebral metastases and glioblastoma (Figures 4A,B), as expected and consistent with other studies (15). Significant positive correlations between the True (favorable) response rate and free-water ratio on the items assessing whether the enhancement provided further clarification into the lesion definition (*r*_s_ = 0.9) and brain-tumor interface (*r*_s_ = 0.68). The majority of surgeons, for example, showed favorable responses to these survey items for corrected tractography in patients 10 and 5 with histopathologies of glioblastoma (S10) and cerebral metastases (S5 and S6) (Figures 4A,B).

**Table 3:** Comparison of differences in response probabilities between standard (uncorrected) and enhanced (corrected) tractography datasets.

Survey item	Un-corrected tractography			Free-water corrected tractography			Stuart-Maxwell test	
	True (%)	Partially true (%)	False (%)	True (%)	Partially true (%)	False (%)	*χ*2 (dof)	Value of *p*
False signal: false tracts are seen in sulci or ventricles (CSF spaces)	13 (15.29%)	21 (24.71%)	51 (60%)	39 (46.99%)	34 (40.96%)	10 (12.05%)	38.93 (2)	<0.001
Grey-white matter interface: the tracts appear to begin at the grey white matter interface	51 (60.71%)	31 (36.9%)	2 (2.38%)	53 (62.35%)	17 (20%)	15 (17.65%)	13.62 (2)	*p* < 0.01
3D-rendering: this scan demonstrates the following anatomical effects: tract reduction	52 (61.9%)	18 (21.43%)	14 (16.67%)	27 (32.53%)	23 (27.71%)	33 (39.76%)	21.38 (2)	<0.001
3D-rendering: this scan demonstrates the following anatomical effects: tract displacement	64 (75.29%)	17 (20%)	4 (4.71%)	67 (78.82%)	15 (17.65%)	3 (3.53%)	0.81 (2)	1
3D-rendering: this scan demonstrates the following anatomical effects: tract infiltration	26 (30.59%)	26 (30.59%)	33 (38.82%)	44 (52.38%)	15 (17.86%)	25 (29.76%)	18.01 (2)	<0.001

CSF, Cerebrospinal Fluid.

### Optimal surgical pathway

We sought to establish the primary limitation of standard tractography when evaluating the optimal surgical approach; namely, the presence of tract reduction in areas essential to the intervention strategy (Figures 2, 3C). Such false negatives will preclude the valuable clinical differentiation of vasogenic edema from infiltrative tumor preoperatively (36), which may impact the resection margins during surgery. Indeed, this undesirable feature of tractography was confirmed with rater agreement that (1) tracts were missing in the vicinity of the lesion and (2) that standard tractography demonstrated tract reduction (Table 2; Figure 3C). Despite false negatives, however, raters strongly agreed that the optimal surgical approach was clear – ascertaining its value as a clinical adjunct. Importantly, a presurgical plan is optimal insofar as its intraoperative execution minimizes postoperative deficits within the desired onco-functional tradeoff. The perilesional sparsity of tract-reconstructions would undoubtedly contradict this objective. When asked whether the enhanced data further clarified the optimal surgical approach, a positive correlation (*r*_s_ = 0.63) was observed between the True response rate and the free-water ratio in the dataset (Figure 4C). This pattern is unsurprising when considering enhanced datasets with less edema will resemble their standard counterparts and when they diverge in their edema extent, the benefits of the enhancement are emphasized – providing evidence that a correction is best applied on select pathologies (i.e., not a one-size-fits all approach).

### Mitigation of false positives

Although advanced diffusion modeling offers insights absent with conventional processing pipelines, these benefits are often counterbalanced by the introduction of false positives (i.e., spurious tracts) (15, 37–39). In the present survey, this trend was noticed by raters particularly along the grey-white matter interface and CSF spaces (Figures 3AB) - underscoring the need to mitigate such artifacts for surgical planning. Together with the understanding that peritumoral diffusion properties vary across unique brain tumors and patients (15), we introduce a novel free-water-parameterized tractogram and thresholding technique which allows for individualized corrections to be applied by the surgeon’s discretion (based on US patent no. 11,355,230) (27). This novel methodology is demonstrated within the edematous zones of a metastatic lesion and in gliomas (Figure 5), where the varying subcortical effects of infiltration, mass effect, and brain-tumor interface can be inspected with progressive changes to the correction strength. Our preliminary observations while using this technique have demonstrated that false positive segments often occur in CSF spaces with consistently high scoring free-water parameters, making the FWC slider an effective filtering tool. Moreover, when the slider is combined with automated methods for clustering tracts of interest, the negative impact of artifactual tracts are further diminished by the anatomical constraints imposed at the clustering step. Nevertheless, the thresholding and tract visualization process for edematous pathologies should be guided solely at the surgeon’s discretion to evaluate the sensitivity of the algorithm within the context of the surgical intervention being planned and the patient’s functional status prior to surgery (40, 41).

### Limitations

The histopathological heterogeneity in cases selected for this analysis were chosen to highlight the variation in edematous zones across a spectrum of neuro-oncologic pathologies and the perceived clinical value of the correction algorithm therein. Given the limited sample of brain malignancies evaluated here, future studies are needed to systematically assess the differential patterns of peritumoral tractography both within (e.g., low- vs. high-grade glioma) and between distinct tumor types (e.g., glioma vs. cerebral metastasis) to ascertain the diagnostic value of this novel tract-based imaging data. Moreover, the benefits of the enhancement for selecting the optimal parafascicular surgical approach must also be further validated against postoperative patient neurological outcomes. With the software implementation of the adaptive thresholding technique presented in this work, such hypotheses can be readily tested with clinically feasible diffusion imaging acquisitions.

## Conclusion

In this work, we implement a novel and noninvasive tractography assessment survey to qualitatively validate multi-subject free-water corrected tractograms along clinically relevant parameters across a variety of edematous pathologies by neurosurgical oncologists. We report that the perceived clinical benefits of free-water corrected tractography are pathology-specific, with favorable assessments of lesion margins, the brain-tumor interface, and optimal surgical pathway being positively correlated with the increasingly edematous datasets consisting of brain malignancies. Surgeon feedback from this novel survey was incorporated into a commercially available software implementation to introduce a first-of-its-kind adaptive FWC tractography thresholding method which may accommodate variability in peritumoral edema effects across patients and pathologies, thereby maximizing its potential as a clinically informative adjunct in otherwise challenging datasets.

## Data availability statement

The MRI datasets presented in this article cannot be made available, however survey response data supporting the conclusions of this article can be made available by the authors. Requests to access the datasets should be directed to WH, Wes.Hodges@SynaptiveMedical.com.

## Ethics statement

Ethical approval was not required for the studies involving humans because this study surveys surgeon perspectives in previously treated neurosurgical patients with anonymized radiographic images. The research did not include interaction or intervention in human subjects. The studies were conducted in accordance with the local legislation and institutional requirements. The human samples used in this study were acquired from a by- product of routine care or industry. Written informed consent to participate in this study was not required from the participants or the participants’ legal guardians/next of kin in accordance with the national legislation and the institutional requirements.

## Author contributions

SK: Formal analysis, Methodology, Writing – original draft, Writing – review & editing, Conceptualization, Data curation, Investigation, Project administration, Resources, Supervision. WH: Conceptualization, Investigation, Methodology, Project administration, Resources, Supervision, Writing – original draft, Writing – review & editing, Software, Validation, Visualization. HA: Formal analysis, Resources, Software, Visualization, Methodology, Writing – review & editing. TH: Resources, Software, Visualization, Methodology, Writing – review & editing. PF: Formal analysis, Investigation, Writing – review & editing. VT: Formal analysis, Writing – review & editing, Investigation. GP: Formal analysis, Investigation, Writing – review & editing. KH: Formal analysis, Investigation, Writing – review & editing. IL: Formal analysis, Writing – review & editing, Investigation. ES: Formal analysis, Investigation, Writing – review & editing. PC: Formal analysis, Investigation, Writing – review & editing. DH: Formal analysis, Investigation, Writing – review & editing. BZ: Formal analysis, Investigation, Writing – review & editing. RV: Investigation, Methodology, Software, Supervision, Validation, Writing – review & editing, Formal analysis. VB: Formal analysis, Writing – review & editing, Methodology, Visualization, Writing – original draft.
